# Supplementary material for: Coping, health anxiety, and stress among health professionals during Covid-19, Cape Coast, Ghana
Source: PLoS One. 2024 Jan 29;19(1):e0296720. doi: 10.1371/journal.pone.0296720 (PMC10824413; doi:10.1371/journal.pone.0296720)
Supplement: S1 Data — (DOCX) [file pone.0296720.s001.docx]

   Data (5).sav

**$FL2@(#) IBM SPSS STATISTICS MS Windows 22.0.0.0 r B Y@17 Jan 2119:23:41 AGE Age of participants GENDER Gender of participants CATEGORY‑ Category of Health Profession SECB1A ‑ I do not worry about my health SECB1B $ I occationally worry about my health SECB1C 0 I spend much of my time worrying about my health SECB1D 0 I spend most of my time worrying about my health SECB2A @ I notice aches and pains less than most other people (of my age) SECB2B A I notice aches and pains as much as most other people (of my age) SECB2C @ I notice aches and pains more than most other people (of my age) SECB2D 6 I am aware of aches and pains in my body all the time SECB3A / I am not aware of bodily sensations or changes SECB3B 7 Sometimes I am aware of bodily sensesations or changes SECB3C 2 I am often aware of bodily sensesations or changes SECB3D 8 I am constantly aware of bodily sensesations or changes SECB4A 1 Resisting thoughts of illness is never a problem SECB4B . Most of times I resist the thoughts of illness SECB4C B I try to resist thoughts of illness but i an often unable to do so SECB4D E Thoughts of illness are so strong that I no longer try to resist them SECB5A . I am not afraid that I have a serious illness SECB5B 3 I am sometimes afraid that I have a serious illness SECB5C / I am often afraid that I have a serious illness SECB5D 0 I am always afraid that I have a serious illness SECB6A : I do not have images (mental pictures) of myself being ill SECB6B . I occationally have images of myself being ill SECB6C , I frequently have images of myself being ill SECB6D , I constantly have images of myself being ill SECB7A I I do not have any difficulty taking my mind off thoughts about my health SECB7B G I sometimes have difficulty taking my mind off thoughts about my health SECB7C D I often have difficulty taking my mind off thoughts about my health SECB7D 5 Nothing can take my mind off thoughts about my health SECB8A H I am lastingly relieved if my physician tells me there is nothing wrong SECB8B > I am initially relieved but the worries sometimes return later SECB8C ; I am initially relieved but the worries always return later SECB8D > I am not relieved if my doctor tells me there is nothing wrong SECB9A 9 If I hear about an illness I never think I have it myself SECB9B @ If I hear of about an illness I sometimes think I have it myself SECB9C > If I hear of about an illness I often think I have it myself SECB9D > If I hear of about an illness I always think I have it myself SECB10A E If I have a bodily sensation or change I rarely wonder what it means SECB10B C If I have a bodily sensation or change I oten wonder what it means SECB10C E If I have a bodily sensation or change I always wonder what it means SECB10D A If I have a bodily sensation or change I must know what it means SECB11A > I usually feel at very low risk for developing serious illness SECB11B @ I usually feel at fairly low risk for developing serious illness SECB11C > I usually feel at moderate risk for developing serious illness SECB11D : I usually feel at high risk for developing serious illness SECB12A ' I never think I have a serious illness SECB12B + I sometimes think I have a serious illness SECB12C ' I often think I have a serious illness SECB12D & I usally think that I am seriously ill SECB13A c If I notice an unexplained bodily sensation I don't find it difficult to think about others things SECB13B h If I notice an unexplained bodily sensation I sometimes find it difficult to think about others things SECB13C c If I notice an unexplained bodily sensation I often find it difficult to think about others things SECB13D c If I notice an unexplained bodily sensation I always find it difficult to think about others things SECB14A E My family and friends would say I do not worry enough about my health SECB14B E My family and friends would say I have a normal attitude to my health SECB14C @ My family and friends would say I worry too much about my health SECB14D 5 My family and friends would say I am a hypochondriac SECB15A O If I had COVID-19, I would still be able to enjoy things in my life quite a lot SECB15B R If I had COVID-19, I would still be able to enjoy things in my life quite a little SECB15C Q If I had COVID-19, I would be almost completely unable to enjoy things in my life SECB15D J If I had COVID-19, I would be completely unable to enjoy things in my life SECB16A c If I had developed a COVID-19, there is a good chance that modern medicine would be able to cure me SECB16B g If I had developed a COVID-19, there is a moderate chance that modern medicine would be able to cure me SECB16C i If I had developed a COVID-19, there is a very small chance that modern medicine would be able to cure me SECB16D _ If I had developed a COVID-19, there is no chance that modern medicine would be able to cure me SECB17A 4 A serious illness would ruin some aspect of my life SECB17B 3 A serious illness would ruin many aspect of my life SECB17C ; A serious illness would ruin almost every aspect of my life SECB17D 4 A serious illness would ruin every aspect of my life SECB18A F If I had a serious illness I would not feel that I had lost my dignity SECB18B B If I had a serious illness I would feel that I had lost my dignity SECB18C Q If I had a serious illness I would feel that I had lost quite a lot of my dignity SECB18D J If I had a serious illness I would feel that I had totally lost my dignity SECC1 ` In the past month, how often have you been upset because of something that happened unexpetedly? SECC2 m In the last month, how often have you felt that you were unable to control the important things in your life SECC3 A In the last month, how often have you felt nervous and "stressed" SECC4 d In the last month, how often have you felt confident about your ability to handle personal problems? SECC5 K In the last month, how often have you felt that things were going your way? SECC6 h In the last month, how often have you found that you could not cope with all things that you had to do? SECC7 T In the last month, how often have you been able to control irritations in your life? SECC8 J In the last month, how often have you felt that you were on top of things? SECC9 d In the last month, how often have you been angered becuase of things that were outside your control? SECC10 q In the last month, how often have you felt difficulties were pilling up so high that you could not overcome them? SECD1 I I've been turning to work or other activities to take my mind off things SECD2 R I've been concentrating my efforts on doing something about the situation I am in SECD3 , I've been saying to myself "this isn't real" SECD4 F I've been been using alcohol or other drugs to make myself feel better SECD5 5 I've been been getting emotional support from others SECD6 * I've been giving up trying to deal with it SECD7 ; I've been taking actions to try to make my situation better SECD8 3 I've been refusing to believe that it has happened SECD9 = I've been saying things to let my unpleasant feelings excape SECD10 4 I've been getting help and advice from other people SECD11 B I've been using alcohol or other drugs to help me get through it. SECD12 Q I've been trying to see it from a different light, to make it seem more positive SECD13 I've been criticizing myself SECD14 : I've been trying to come up with strategy about what to do SECD15 = I've been getting comfort and and understanding from someone SECD16 ( I've been giving up the attempt to cope SECD17 : I've been looking for something good in what is happening SECD18 ­ I've been making jokes about it SECD19 I've been doing something to think about it less, such as going to movies, watching TV, reading, daydreaming, sleeping or shoping SECD20 A I've been accepting the reality of the fact that it has happened SECD21 * I've been expressing my negetive feelings SECD22 E I've been trying to find comfort in my religion or spiritual beliefs SECD23 H I've been trying to get advice or help from other people about what I do SECD24 " I've been learning to live with it SECD25 2 I've been thinking hard about what steps to take. SECD26 2 I've been blaming myself for things that happened SECD27 I've been praying and meditating SECD28 % I've been making fun of the situation VAR00001 ?**

**18-29years @**

**30-49years @**

**50-60years ?Male @Female ?Medical Doctor @Physician Assistant @Nurse ?Yes @No H**

**‑ ­ ! " # $ % & ' ( ) * + , - . / 0 1 2 3 4 5 6 7 8 9 : ; < = > ? @ A B C D E F G H I J K ?Never @Almost @ Sometimes @Fairly Often @**

**Very Often**

**L M N O P Q R S T U ? I haven't been doing this at all @A little bit @A medium amount @I have been doing this a lot V W X Y Z [ \ ] ^ _ ` a b c d e f g h i j k l m ? I haven't been doing this at all @A little bit @A medium amount @I have been doing this a lot n o p ? I haven't been doing this at all @A little bit @A medium amount @I have been doing this a lot q **

**V**

**aAGE=Age GENDER=Gender CATEGORY=Category SECB1A=SecB1a SECB1B=SecB1b SECB1C=SecB1c SECB1D=SecB1d SECB2A=SecB2a SECB2B=SecB2b SECB2C=SecB2c SECB2D=SecB2d SECB3A=SecB3a SECB3B=SecB3b SECB3C=SecB3c SECB3D=SecB3d SECB4A=SecB4a SECB4B=SecB4b SECB4C=SecB4c SECB4D=SecB4d SECB5A=SecB5a SECB5B=SecB5b SECB5C=SecB5c SECB5D=SecB5d SECB6A=SecB6a SECB6B=SecB6b SECB6C=SecB6c SECB6D=SecB6d SECB7A=SecB7a SECB7B=SecB7b SECB7C=SecB7c SECB7D=SecB7d SECB8A=SecB8a SECB8B=SecB8b SECB8C=SecB8c SECB8D=SecB8d SECB9A=SecB9a SECB9B=SecB9b SECB9C=SecB9c SECB9D=SecB9d SECB10A=SecB10a SECB10B=SecB10b SECB10C=SecB10c SECB10D=SecB10d SECB11A=SecB11a SECB11B=SecB11b SECB11C=SecB11c SECB11D=SecB11d SECB12A=SecB12a SECB12B=SecB12b SECB12C=SecB12c SECB12D=SecB12d SECB13A=SecB13a SECB13B=SecB13b SECB13C=SecB13c SECB13D=SecB13d SECB14A=SecB14a SECB14B=SecB14b SECB14C=SecB14c SECB14D=SecB14d SECB15A=SecB15a SECB15B=SecB15b SECB15C=SecB15c SECB15D=SecB15d SECB16A=SecB16a SECB16B=SecB16b SECB16C=SecB16c SECB16D=SecB16d SECB17A=SecB17a SECB17B=SecB17b SECB17C=SecB17c SECB17D=SecB17d SECB18A=SecB18a SECB18B=SecB18b SECB18C=SecB18c SECB18D=SecB18d SECC1=SecC1 SECC2=SecC2 SECC3=SecC3 SECC4=SecC4 SECC5=SecC5 SECC6=SecC6 SECC7=SecC7 SECC8=SecC8 SECC9=SecC9 SECC10=SecC10 SECD1=SecD1 SECD2=SecD2 SECD3=SecD3 SECD4=SecD4 SECD5=SecD5 SECD6=SecD6 SECD7=SecD7 SECD8=SecD8 SECD9=SecD9 SECD10=SecD10 SECD11=SecD11 SECD12=SecD12 SECD13=SecD13 SECD14=SecD14 SECD15=SecD15 SECD16=SecD16 SECD17=SecD17 SECD18=SecD18 SECD19=SecD19 SECD20=SecD20 SECD21=SecD21 SECD22=SecD22 SECD23=SecD23 SECD24=SecD24 SECD25=SecD25 SECD26=SecD26 SECD27=SecD27 SECD28=SecD28 VAR00001=VAR00001 B Age:$@Role('0'**

**)/Gender:$@Role('0'**

**)/Category:$@Role('0'**

**)/SecB1a:$@Role('0'**

**)/SecB1b:$@Role('0'**

**)/SecB1c:$@Role('0'**

**)/SecB1d:$@Role('0'**

**)/SecB2a:$@Role('0'**

**)/SecB2b:$@Role('0'**

**)/SecB2c:$@Role('0'**

**)/SecB2d:$@Role('0'**

**)/SecB3a:$@Role('0'**

**)/SecB3b:$@Role('0'**

**)/SecB3c:$@Role('0'**

**)/SecB3d:$@Role('0'**

**)/SecB4a:$@Role('0'**

**)/SecB4b:$@Role('0'**

**)/SecB4c:$@Role('0'**

**)/SecB4d:$@Role('0'**

**)/SecB5a:$@Role('0'**

**)/SecB5b:$@Role('0'**

**)/SecB5c:$@Role('0'**

**)/SecB5d:$@Role('0'**

**)/SecB6a:$@Role('0'**

**)/SecB6b:$@Role('0'**

**)/SecB6c:$@Role('0'**

**)/SecB6d:$@Role('0'**

**)/SecB7a:$@Role('0'**

**)/SecB7b:$@Role('0'**

**)/SecB7c:$@Role('0'**

**)/SecB7d:$@Role('0'**

**)/SecB8a:$@Role('0'**

**)/SecB8b:$@Role('0'**

**)/SecB8c:$@Role('0'**

**)/SecB8d:$@Role('0'**

**)/SecB9a:$@Role('0'**

**)/SecB9b:$@Role('0'**

**)/SecB9c:$@Role('0'**

**)/SecB9d:$@Role('0'**

**)/SecB10a:$@Role('0'**

**)/SecB10b:$@Role('0'**

**)/SecB10c:$@Role('0'**

**)/SecB10d:$@Role('0'**

**)/SecB11a:$@Role('0'**

**)/SecB11b:$@Role('0'**

**)/SecB11c:$@Role('0'**

**)/SecB11d:$@Role('0'**

**)/SecB12a:$@Role('0'**

**)/SecB12b:$@Role('0'**

**)/SecB12c:$@Role('0'**

**)/SecB12d:$@Role('0'**

**)/SecB13a:$@Role('0'**

**)/SecB13b:$@Role('0'**

**)/SecB13c:$@Role('0'**

**)/SecB13d:$@Role('0'**

**)/SecB14a:$@Role('0'**

**)/SecB14b:$@Role('0'**

**)/SecB14c:$@Role('0'**

**)/SecB14d:$@Role('0'**

**)/SecB15a:$@Role('0'**

**)/SecB15b:$@Role('0'**

**)/SecB15c:$@Role('0'**

**)/SecB15d:$@Role('0'**

**)/SecB16a:$@Role('0'**

**)/SecB16b:$@Role('0'**

**)/SecB16c:$@Role('0'**

**)/SecB16d:$@Role('0'**

**)/SecB17a:$@Role('0'**

**)/SecB17b:$@Role('0'**

**)/SecB17c:$@Role('0'**

**)/SecB17d:$@Role('0'**

**)/SecB18a:$@Role('0'**

**)/SecB18b:$@Role('0'**

**)/SecB18c:$@Role('0'**

**)/SecB18d:$@Role('0'**

**)/SecC1:$@Role('0'**

**)/SecC2:$@Role('0'**

**)/SecC3:$@Role('0'**

**)/SecC4:$@Role('0'**

**)/SecC5:$@Role('0'**

**)/SecC6:$@Role('0'**

**)/SecC7:$@Role('0'**

**)/SecC8:$@Role('0'**

**)/SecC9:$@Role('0'**

**)/SecC10:$@Role('0'**

**)/SecD1:$@Role('0'**

**)/SecD2:$@Role('0'**

**)/SecD3:$@Role('0'**

**)/SecD4:$@Role('0'**

**)/SecD5:$@Role('0'**

**)/SecD6:$@Role('0'**

**)/SecD7:$@Role('0'**

**)/SecD8:$@Role('0'**

**)/SecD9:$@Role('0'**

**)/SecD10:$@Role('0'**

**)/SecD11:$@Role('0'**

**)/SecD12:$@Role('0'**

**)/SecD13:$@Role('0'**

**)/SecD14:$@Role('0'**

**)/SecD15:$@Role('0'**

**)/SecD16:$@Role('0'**

**)/SecD17:$@Role('0'**

**)/SecD18:$@Role('0'**

**)/SecD19:$@Role('0'**

**)/SecD20:$@Role('0'**

**)/SecD21:$@Role('0'**

**)/SecD22:$@Role('0'**

**)/SecD23:$@Role('0'**

**)/SecD24:$@Role('0'**

**)/SecD25:$@Role('0'**

**)/SecD26:$@Role('0'**

**)/SecD27:$@Role('0'**

**)/SecD28:$@Role('0'**

**)/VAR00001:$@Role('0'**

**) UTF-8 feefeffefffffefefffeeffefffefffefffeeffefffeeffefffefffeeffeeffefffeeffeeffghgghegiggeffeeeefefeefgeefegfegegfege ffffeffeffffefffeffefffefffefffefffefffefffefffefffefffefffeffffeffefffefffeegfgeigeeeeeefeeeeeeeeeeefffeeffffege eeffefffeffffeffefffeffefffefffeffffeffffefefffefffeffffefffeffeffffeffefffgggifgihihhhefeegehffgehfegeegeeeegfee fefeffffeffeffffefffefffefffefffefffeffeffffeffeffffefffefffefffeffeffffeffgfgfgfgfgfgfgfgfgfgfgfgfgfgfgggggggggg effeffffeffffefffeffeffefffffefffefffeffefffefffeffffeffeffefffeffffeffffefefgfggfefgegfgfgfgfegefgefefgfegefefgh fefeffffefffefffeffeffffefffeffeffffefffefffefffeffeffffefffeffeffffefffeffgfgfgggfgfgfffgefgfgffggfgfgfhgfgggggg eeffeffeffffeffffeffefffefffefffffefeffeffffeffeffffeffefffeffffeffefffefffhgghghgfgggfeegfgegfgfgegfgegfggffgegf ffggeffffefffefffefffeffffeffefffeffeffefffefffeffffefffeffeffffefffefffeffggggggfgfggggeggfgfggfgfgfgegffgefgfeg eegfffeffeffeffffefffeffeffeffffefffeffeffffefffeffeffffeffefffefffeffffeffgfggfggfgfgfgfgegfgfgfgeggegfgfggggfgg ffgeffffefffeffffefefffefffeffffeffefffefffefffefffefffefffeffeffffefffeffffgfgfggfgggfgfgfggfggfgfgfgfggfgffggfg ffgfefffefffefffefffffefffeffefefffffefeffffefffffeeffffeffefffeffffefffeffggggggfgfggfgfgfgggggfgfgfgfgfgggggggg ffgeffffefffefffeffefffeffffeffefffefffeffffefffefffefffeffeffffefffefffeffggggggfgfggghegfhfegffeggfgfgfgggfgfgg ffgfefffefffefffffefefffefffefffeffeffffefffeffefffeffffeffefffefffefffefffgggfggfggfegegfhfhgggehhgfhgheghfghehh gfgefffeffffefffefffeffffefffefffeffeffeffffeffeffffeffefffeffffeffeffffeffgfgfgggfgggfghegfgfghghfgghhgehggggfgg efgfefffefffeffffeffefffefffeffeffffefffefffefffeffeffffefffefffefffefffeffgfgfgfgfghgfgfgfegfggfgehgfggfgfggfgfg eegfefffefffefffffeffeffefffefffeffeffffefffefffeffeffffefffeffefffeffffeffgfgfggfggfggfggfgfgfgggfgfgfgfgfgggfgg efgfffeffefffeffeffffeffefffeffeffffefffefffefffeffeffffefffeffeffffefffeffgfgfgggfgffgfgfgfgfggffgfgegegfgggggfg gfgfefffefffefffefffeffffffffffefeffeffffeffefffefffffeffeffeffffeffefffeffgggggghghhghghghghggggggfgghhgghghfhgg fggfefffeffffefffefffefffeffeffffefffeffeffeffffefffefffeffefffeffffefffeffgfgfgfgfggfgggfgfgfggfgfgfggggefggfgfg efgfeffeffffefffffefefffefffffefefffefffeffffefeffffefffeffefffeffffefffeffgfgfgffgghgfgfgfgfhggfgfgfgffggfggffef efgfefffeffffeffffeffefffeffeffffeffeffeffffefffffefeffffeffffeffeffefffffehghgggggfghgggfgggfgfgfgfggghggfgfggfg efgeffffefffefffeffffeffeffefffeffffeffefffefffeffefeffffeffefffeffeffffeffefgfgfgefffefegfegfgegfeggfgegefggfefg eegfffefefffffefefffffefefffefffeffffeffefffefffeffefffeffffefffeffefffefffegffggefggefgggegeffefgegefffefefffgfe efgfefffefffeffffeffffefffeffeffeffffefffeffffefefffeffffefffeffeffeffffefffghgggggfgghghgeggggggfgfghfgffggfhfgg fegfffefeffffefeffffefffefffefffeffefffeffffefffeffffefefffefffeffffefffeffeefhgfgfgffghffgfgfgeggfgfgfhefgggfgfg ffgfefffefffeffffeffefffeffffefffeffffeffeffefffefffefffefffefffefffeffffefgfggfgggfhghfgfgegfggfggffgeggefgfggfg ffgfeffffefffeffeffffefffefffefeffffefefefffefffefffefffefffeffeffffefffeffgfggfgffgggfgfgfgfgggghfgggfhffggfggfg ffgeffffefffefffefffefffeffffeffefffeffffeffefffffeffeffeffeffffefffefffeffgfgfgggfgfgfgfgfgfgggfgegfggegfgggfgfg ffgeffffeffffefffeffefffefffefffefffefffefffefeefffeffffefffefffeffeffffeffgfgfggfggfgegfgffggggeegfegeggfggffhhh efgeffffeffffefffeffffeffeffefffefffeffefffeffffefffefeffeffffefefffefffefffgfgfgfhffeghghgfeeffefgefegefgggegeef efgffefefffffeffeffeffffeffefffefffeffffeffefffeffffefffeffefffefffefffefffggfgfffgegfgffgffeffehfgeghefgfhffhgfg efgfefffeffffefffeffeffeffffeefffefefffefffeeffefffefffffefeffffeffeeffefffghigghhfiiggeeffgegeefeggffefgfggfffhf efgfefffefffeffeffffefffefffeffefffeffffefffefffefffefffefffeffeffffeffefffgfggggfggggfgheggefggfgfgffgfggfefgffg efgfeffeffffefffefffefffefffefffffefeffefffefffeffefeffffeffefffefffefffeffgfgfgfgfgggfgfggggggfgggfgfgfgfgggggfe ffgffeffffeefffeffffefffefffefffffefffeffeffeffefffefffeffffeffefffefffffefgfgfgffggggfheggfgfggfgfefgfegfegggfgf efgfeffffefffefffefffefffeffeffffeffefffefffefffeffeffeffeffefffefffeffffefggfggggfgfgfgegfgfgfgfghfggfgfgfggfgfg ffgeffffefffefffffeffefffeffefffeffffefffefeffffeffeffffefffefffeffffeffeffggggghgfgggfggfgfgfggfggfgfgggfeeggfgg efgffefffefffeffffefefffeffefffffeffefffefefefffeffefffffeffffeeffffefffeffgfgfgfgfhfghfgfghfgfgfggegegfgefggggfg ffgeffffefffefffefffeffefffeffffeffefffefffeffffefffeffefffeffffeffffeffefffgggfgfgfggfegfggfgfgffgfgfgggggggggfe ffgffefefffeffffefffefffeffffefffeffffeffeffeffffeffefffeffefffeffffefffeffgfghghfgfggegfgefggfggfefgfgfgefggfgfg efgfeffffefffefffefffeffefffefffeffffeffefffefffeffeffffefffefffefffefffeffggfgfggfgfgffggfgfgfgfgfgffggfgfggfggf efgffefffeffefffefffefffeffffeffffeffeffefffefffefffefffefffffefefffefffeffgfhgfgfgfggfgeegegfggfgefgegffgeggegfg efgfeffeffffeffffefefffefffeffffeffeffffeffefffeffffefffeffffefefffefffefffgeggfgggggffffffffffeegegggggggggggggg efgfffeeffffffefefffeffeffffeffefffeffffffeefffefffeffffefffeffefffefffefffgfgfggfgghefefffefffefggeffgfffggggggg efgfffefffeffefffefffefefffefffefffefffffefffeffffeffefffeffefffeffffeffeffggggigfggfffeegefgfgfgfggfeffffffgffgh eegfffeffeffeffefffefffffeffeffffeffeffeffeefffeffffeffeffffeffefffefffefffefggfggfgfegggfgffgefegfheffffgfffgfgg ffgffefffefffefffefffeffefffefffefffefffefffeffeffffefffefffefeefefefffefffefefefefeefeefeefeeeeeeeefffggfgfgffff efgffeffefffefffefffeffeffffefffefffeffffefffeffefffefffefffeffffefffefffefggghgghghefeeffgeefegefgefffffffffgfgg efgfeffeffffefffeffeffffeffefffefffeffffeffefffeffffefffeffefffefffefffefffeeeifeffgehhhhhhhhhhehehfehfehhhhhhefe ffgfeffffefffeffffeffefefffefffefffeffffeffefffefffefffefffefffeffffffeefffhfffhiehegfeeehefefhhegeeheggheeehgfee eegfefffeffffeffefffeffeffffeffffeffefffefffefffefffefffefffefffefffefffeffhghghhghhggggfgggfgggfgggfggfggfgggggf ffgfefffeffeffffffefffeffeffeffffeffefffefffefffefffefffefffefffefffefffeffgfgfgfgfgfgffgggefgggggffegegfgefgfggg efgeffffeffffefffefffefffeffeffffeffffefeffffeffeffeffeffeffefefefffefffefffgggfgfgfghhhehehghhehfhhghfhfggfggggg ffgfefffeffffeffefffefffefffefffeffefffffefffeffefffefffeffffefefffffefeffffefiheiheeeheegeheegeeeefeheehfhfhhege efgfeffffefffeffffeffeffefffeffffeffeffffefffefefffeffffefffefffefffefffeffgfgfggfgfghgfgefgegfgfgfgegffgffggfgfg efgffeffefffeffefffffeffeffffeffeffffefffefffeffeffefeffefffffeffefffeffeegfgfgfgfhgfghegegfgfeghfggefgfgeggggfgg efgfefffeffffefffeffefffefffefffffefffefefffefffeffeffffefffefffefffeffffeffgfgfgfgfggegegfggehfgfggfgfgfgfggggfg eegfeffffeffeffeffffeffffeffeffefffffeffefffefffffeeffffefffeffffeffefffeffggfgfggfgfgffgefefeggegfgegeggegeegfgf efgffeffffeffefffeffefffefffefffefffeffffeffefffeffffeffefffefffeffffefffefghfgfehhghggfhgfhgfhfhfgfffhgfefhhgfgf eegfefffffefeffffeffefffffeffefffefffeffefffefffffefefffefffefffefffefffffegghhhghgigeeeeeeeeeeeeeegeeeeeeeeeeeee eegfefffeffffeffefffeffefffefffeffffeffefffffefffeffefffefffeffefffefffefffghfgfegeffgefhgffghhefffgghfefhegeffgf fegfeffffefffefefffefffefffefffffeffeffffeffffeefffefffeffffeffefffeffffffeeeghhggeeeeeefeefeefeeeeffefefehfhffff efgffefefffffefeffffeffefffeffffeffeffffefffefffeffefffefffefffffefefffefffeegeheheeeegeheeeegeeeegegeegehehehege ffgeffffffefffeefffefffefffeffffeffeffffffefefffeffeffffefffefffefffefffeffggeigegggiefffghggfggeeefhggfeggehgfhh efgeefffefffeefeeffefffefffeffffefffefffefffeffeeffeeffeeffffeffeffeffffeffhghigghhgghheegfhefgefgggeffghghhghghe ffgfffeeffffffeefffefffefffeffffefffeffefffefffeffffefffefffffefeffefffefffgghiggigeefhfggehfhhehehgehhhhfhfhffhh efgefffeffffffeefffefffeffffffeffefefffefffefffeffffefffeffefffffefeffffeffegggeegggghhgfgghehfeheehehehehgehhhhh efgfffefefffefffefffefffefffefffeffeffffeffefffefeffeffefffeffffeffeffffefffggegeggggffeeefffffffeffffffefffeffhf efgfffefffefffefffeffefeffffffefffeefffffeffffeefffeffffeffeffffeffefffefffggfifeeifeheeeefeehhhehhgehefhgeegeeef eegfeffefffffefeffffefffeffffefffeffefffeffefffeff effffeffefffffefffefffefhegfiggfhghgfefgegggehfhgfhhhhgfehgfeg efgffeffefffeffzefffefffefffeffffefffeffffefffeffefefffeffffffeffefffefffefefeefeefeeeffefeeeeeeefeeefeefeeeeeeee efgfeffffefffefffefffeffeffffeffffefffeffefffefffefefffffeffefeffefffefffefgfgffgffffffegefegeefeegeggeefeeegeefg eegfeffefffffefeffffeffefffefffeffffefffefffefffeffeffffefffeffeffffeffeffffgfihhihggegeefegeegeehfegeehffgghhefe efgfffefffefeffffeffefffefffefffeffffeffffeffeffeffefffffeffefffeffefffefffgeghgggggghgeehghhhhehghhfghhhhhhhhhgg efgfefffeffffeffefffefffeffefffefffefffeffffffeefffefffffefefffefffeff feffigiighgghfggfefehhffegehfggfghfffggffe efgfffeefffffefffeffefffefffefffefffefffeffeffffefffeffffefefffefffeffffeffgfffhghheffegefeffghefhegeehgffeheegeg ffeeffffeffffefefffeffffeffeff efffefffeffffeffeffffefffeffefffefffefffefffgegffeffeefeeeeeheeeehfhffhgfgeffffegg ffgfefffefffffefeffefffefffefffefffefffefffffefefffeffffeffefffefffefffefffgigiheiihegheeheheghehehgehgfhghhhgfhf efffffefffefffefffefffefffefefffefffeffffeffffeffeffeffffeffffefeeffffeffefgighghghgggfhgfggfghghfgfgfgggggfgfhge eegffeffffefffeffeffeffeffffeffffefefffeffffefffeffffeffffefffeffeffffefefffeiffhhfehfhfeeghfefefeefhffefeffehghe ffgfffeeffffefffefffeffefffeffffeffffeffeffeffffefffeffffefefffeffffffeefffgfgiggggggfgfegghhhheheggehegheegfghhe fegfefffefffeffffeffefffefffefffeffeffffefffffeeffffeffefffffffefffffeffffegfggggggggfggefgggfgeggggggeeegegggege fegfffeffefffefffefffefffefffefffefffefefffffeffffefefffffeeffefffeffeffeffffggefgfggfheehhhgfggfhgfegghggeffgfgh efgfefffefffefffefffeffefffeffffeffefffeffffffefefffefffefffefffeffefffefffgggggggggghfgefegfgfegfgfegghgfgggfffg efgfefffefffeffefffffefeffffeffeffffeffffeffeffefffffeffeffffefffefefffefffgegffgghgefgeegeheggegehhegefgghhgfehe ffgfffefeffffefeefffeffefffeffffefffeffffeffefffeffefffffeffeffefffffefefffiggghggfhghgeegehefhhegeggeggghhhhgehe ffgfeffefffffeffeffefffefffefffefffeffffeffefffefffeffffeffeffffeffeffffeffgeghhhghgeeheefehefgegfhfeffehffgeghef efgfefffefffefffeffefffefffeffffefffefffeffefffefffefffeffffeffefffefffefffggggggggggffffffffffefefffffffffffhhhe fegfeffffefefffffefefffefffefffefffefffefffefffefffeffffeffffeffefffefffeffeghghghhfgghfeeeeeeeeeeeeeeeeeeeeeeeee fegeeffefffeeefeeffeeffeeffeeffeeffeefffeffefffefffefffefffeeefefffefffeefffefefefffffhfegehfeeeffhheeheffheefhfh ffgfeffefffeffffefffeffefffefffefffefffffefefffefffffeffeffefffefffefffefffhegiihhieeeffegehheffefehfehehgehhhehf eegfeeefffefefffffeefffefffefffefffeffffeffefffefffeffffeffeffffeffefffefffgefghihefhhhfefggehheghgffeeghhehghhee fegffefefffeffffffeffefffeffeffffeffffeffefffefffeffefffffeefffffeffffeffefhfggggfggggfffggghfffgfgfeggggegefgfgg ffgefffffeffefffeffefffefffeffffffeeffffeffefffeffffefffeffffeffefffffefffeghihfggfhhhhfegghfffegggfggfggffgggffe ffgfefffeffffefffefffefefffefffefffeffffeffffeffeffefffffefffefffeffefffeffhghggfgggggffegffgfghgeffffeffeefffeee efgefffffefefffffefeffffeffffeffffefeffffefffefeff effffefffefffeffefffeffffgggggffffeeegfhhggfeeffegfghgffgeeeee efgffefeffffeffeffffeffefffefffefffeffffefffeffefffeffffefffefffefffefffeffgegigegggeeeeeeeeeeeeeeeeeeeeeeeeeeeee efgfeffeffffffeeffffefffefffefffeffeffffffeefffeffffffeffeffffefefffffefffehggiiiiighghhehghhhhehhhhghhhhhghghghe efgefffeffffeffffeffeffffeffffefffeffeffefffffeffeffeffeffffeffffeffefffeffgggggggeeefgeegfeghgfegegeeghgggfeeffe eegffeffffefffefffeffeffefffefffffeffeffeffffefffeffeffffeffefffffefffefffefhfiggfgghghgeggefegghgggghfghehghhheh efgfeffffeffefffefffeffefffefffffeffeffffeffffefefffeffefffffffffeffeffffefggggggggggggggggggfggfgfggffgggggggggg efgfefffefffefffefffeffefffefffefffefffefffefffefffefffefffefffefffefffefffeeeeeeeeeeeeeeeeeeeeeeeeeeeeeeeeeeeeee efgfefffffeefffeffffffeefffefffffeffffefffeefffeffffefffefffefffeffeffffeffghfffggfhghffefghefgheheeeefgfghhffefe eegffeffeffffeffeffefffefffeffffffeeffffffefffeefffefffefffefffefffeffffeffhfgfhigigfhfgheghehehfgehfhegefheefgeg ffgffefefffffefefffefffefffefffefffeffffffeeffffffeeffffeffefffefffefffefffggiiigiigghhhehhhhhhhehehhhhhhhhhhhehh efgefffeffffeffefffefffefffefffefffeffffefffeffefffefffefffefffefffefffefffghiggggghhhheegehfegegfggfgghgghefgfhg efgffefeffffefffeffefffeffffeffefffeffffffeefffefffefffffefeffffffeefffefffgeefgfffhheehegefhghefehgegfhgfhhhgghf efgfffeeffffffefefffffeefffffefefffeffffffeefffeffffffeffefffeffffefffeefffiiiigiiiihhhhegfhhhgehhggfgeghfhgghhhe efgffeffffefefffeffeffffffeffefefffffefffefefffeffffefffefffefffeffefffefffieiiieiiiihhefehefehhhfhhhfhehfhfhhfhh efgffeffffeffeffefffeffefffefffefffeffffffeeffffefffffefefffffeefffffeffeffgegiggfhgfhgfefffefgegfhffheggggffhghe efgffeffffeeffffeffffeffefffefffeffefffefffffefefffeffffeffefffeffefefffeffhghhghhhihhheehhhgggehhgfghhhghghhghgg efgfeffefffffeffefffeffeffffffefffefffefefffefffefffeffffeffeffffefefffefffiggghehfgeehehfeefggeegghhfgeggegehehf fegfefffffeefffefffefffeffffffeefffeffffffeffefeffffeffffeffffeeffffffeffefefeghiehgffhegffgfgffeghgehfgfgfghgfgh efgffefeffffeffefffefffefffefffefffeffffffeefffeffffefffeffefffefffefffefffeeifggfgheeffeeehfefefffeffffffffefefe efgfefffefffefffefffeffefffeffffefffefffefffefffffefeffeffffefffeffeffffffefhhgfghhfhgggeeegffeegegeegeefffeggege efgfffeffefffeffefffefffeefefffffefffeeefffefffefffefffffeffefffeffffeffeffeggggfffhgefgfgegfgfefefeggfgefgeefeff efgfeffeffffeffeffffeffefffefffefffeffffeffefffefffefffeffffefffeffefffefffgfgihehigghheehehehhehehheghhhhhhhhhhh efgfeffefffffeffeffefffefffefffeffffeffeffffeffffeffeffeffffefffeffffefefffgegfgffeeeefffefefefegegegeggeffffefef efgfeffffeffeffefffefffefffefffeffffefffeffefffefffeffffefffeffefffefffefffgegiigffhheefegggfeeeeefeegeefeeegfehe efgfefffeffffefefffffefefffeffffeffefffeffffeffeffffeffefffffeffefffffefeffgggghghieeffeeeegeegeeeeeeeeeeeeeeeeee efgffeffffefffefeffefffeffffefffeffeffffeffffefefffffeffefffeffefffefffffefhehiieihiehheehehefhefghhehefhghhghhhe ffgfffefeffeffffefffeffeffffeffefffeffffffefeffefffeffffeffefffefffeffffeffegfiigghggeheehhhehhehehhehfhhhfhhheff ffefeffffffefeffeffffeffeffefffefffeffffeffeffffefffefffefffefffeffefffefffhehgggfggefheegehffgeffhfegefgfffggfff eegfeffefffffeffeffeffffeffefffeffffefffeffeffffeffffeffefffefffeffefffefffhhggggigggfgeegffffgeffhfegffgfffggfff efgfeffeffffffeefffefffefffeffffeffeffffeffefffeffffefffefffeffffefefffefffhggihighhgfhgeegfgfeeheggehgegeheeeehe efgfefffefffefffefffeffeffffeffeffffefffeffffefffeffeffeffffffefefffefffeffghifghgfhhfgeegfgffgegfggfgffggfggffee efgffeffeffffeffeffffefefffefffefffffefffeffefffefffefffeffefffefffefffefffgigihheghggeeeeeeeeeeeeeeeeeeeeeeeeeee efgfeffffefffefffeffffeeffffefffeffefffeffeffeffeffefffeffffeffefffefffefffeggghegggeegghegghegghfghegggegggefhgf efgeffffeffffefffefefffefffefffffefffefffeffeffefffeffffeffefffefffffefefffgheigfgfeiehffgeggeggfefhggffffeefgfee ffgeffffeffefffhfefffeffefffefffffefefffeffffefefffefffeffffeffefffefffefffefeggfghhgehfgheehehgffgfggfegefgehghg efgfefffefffffeffefefffeffffeffefffeffffefffffeeffffeffffeffeffefffefffefffehfigehheeegfeeefegfeeeehehhehfhehhegh eeffeffeffffffeeffffeffefffefffefffeffffeffefffefffeffffeffeffffeffeffffefffgghhfhhhihhhegehhhfehhhgghhhhhhfgggee eefeffffffefffefeffffefffeffefffeffefffeffffeffffeffefffefffeffffefffefffeffggihgegfheegfhfhfghefeggfgfgeefghgffe eegfefffefffefffefffffeefffeffffeffeffffefffefffeffeffffeffefffefffeffffeffggghhghhgeehfeeehfehefeffehfghefhffehe efgfefffefffffefefffefffeffefffeffffefffeffffeffeffeffffeffefffefffeffffeffggghgfhghghhgeheggehehehgehghhehghgehh ffgfefffefffffefefffeffeffffeffefffeffffffeefffeffffefffeffefffefffefffefffighhighhhhghgegghgggegeggegggggggegegg efgfefffffeffefffeffefffeffffefffeffefffeffffefffeffeffffefefffefffeffffeffghgghfhggeegfgfgfffegffgfeffgegfffffeh ffgfefffeffffeffefffeffeffffefffefffefffeffffeffeffffefeffffffefeffefefefffgfghgghghghgfegegefgegfghegfgffggfgefe efgfeffeffffffeefffefffefffefffefffefffefffefffefffeffffeffefffefffefffefffgeeefeeeeeeeeeeeeeeegeffgeggggggghgfgh eeefeffefffeffffefffefffeffefffeefffefffefffeffeffffefffefffeffefffefffefffghghhgfhgffgeeffffgfegegfeggfffgfggfgg efgfefffffefffeffeffeffefffefffefffeffffeffeffffefffefffeffffeffefffeffffefigiggifggihhgegfhghheheghehhhhghhghfhh eeefefffefffefffefffefffeffefffeffffefffeffefffeffffefffeffeffffeffeffffeffigihfgigfggfeegfgfegfgegegghfegehhgeef eeeeffffffeefffffefefffefffefffeffffeffeffffeffefffeffffeffeffffeffefffeffffhifghifeihgegfhgefheheffegehhfgggfefe eeefefffefffefffefffeffefffffeffeffeffffefffeffeffffefffefffefffefffefffeffehgfiffghghggeghfeghegegfhhegfeehghefe feffeffeffffefffeffefffeffffeffeffffefffffefffeeffffefffeffffefffeffefffefffeghgfhghhgfeegefhhgfhhggfgehgehhghhge fefeffffefffeffffeffeffffeffefffefffefffefffeffffefffefffefffeffeffffefffeffeehhfiigfhgfegfghehegefgfhegfehggfghe feeffeffffefffeeffffefffeffeeffeeffeffffffeeeffeffffefffefffeffefffefffefffgefhhggihhehfegehfgfegghgfggfgggheeeee eeffffeffeffefffeffffefffefffefffeffeffefffefffeffefffffefffeffefeffeffffeffgfgifgfhgfggfhhfgghgggfgfgfhfgefgggfh efgfeffefffffefffefeffffeffefffefffeffffeffefffefffeffffefffefffeffefffefffhefiheiheeehheegehefhegehgehghhggfhfef ffefeffeffffffeffeffefffefffefffefffeffffefffeffefffeffffeffeffefffeffffeffhgifggfheehheeefhhhffhfhfehfhhhheffhee ffgefffffeffefffefffefffefffefffeffffeffefffefffefffefffeffeffffefffefffefffgfgfggfgfgfgegfgfgggegfgfgfgfggfggfgg efgfeffffefffefffeffeffeffffeffefffeffffefffefffefffeffffefefffefffffeffeffeffhiehhefffeefeeeffefegfffgggffffgfff eegfffefffefffefffefffefffefffefffeffefffefffeffffefeffffeffefffeffffefffefgfghghffefhhheggffggfggggghggfefhhhhhh ffgfeffeffffeffefffefffefffefffefffefffefffefffefffefffeffffeffeffffeffefffhhihfhhhiigggefghfggegffffgfgfgggfgggg efgfefffeffffefffefffefeffffeffefffeffffeffeffffefffeffffefefffeffffeffefffiiggihhhggfhfeeefgffefeffeghffffffgeff ffgfefffffeefffefffefffefffeffffffeeffffffefeffeffffefffefffffefffefffefeffghgfiffgeeffffffffffffffffffffffffffff efgffeffeffffefffeffffefefffeffffeffefffeffffefffeffefffeffeffefeffffefffefghgffghgfihfggfhgfhgggghffegffgeggfgfh ffgfefffefffefffefffeffefffeffffefffeffffefefffefffefffeffffeffeffffeffffefgfgfghffhfhgfegegehgeghggegfggghghhhhg efgfffefffefeffffeffefffefffefffeffffeffffeffeffeffefffffeffefffeffefffefffgeghgggggghgeehghhhhehghhfghhhhhhhhhgg efgffeffeffffefffeffeffeffffeffefffeffffefffeffefffeffffefffeffefffefffefffghfiighhfgfgeeeeheeeegfheegefgffehfeee efgfffefeffffefffefefffeffffeffefffefffffeffefffffeffeffefffeffefffefffefffffgiighifffgeeeegeeeegeheegehhefeggeee efgfeffeffffeffffeffefffefffefffffefffeffefffeffffeffefffefffffefeffffefffeffhefifggggffgefehggfefeffegeeefffefee efgfefffefffeffeffffeffeffffffefffefeffeffffffefefffefffefffeffffeffefffeffghfghghghhfhffffgeffeffghggfhfhhhhhhhh fegfefffefffefffefffefffefffefffeffefffefffefffefffeffffeffeffffeffeffffeffiihgggffifefeeeeeeeeeeeeeefffeeeggeeee ffgfefffeffeffffeffeffffefffefffefffffefeffeffffeffeffffeffeffffffeffefffefeffggffgfiefhggfehheeffgfegeggeffehhfh efgfefffefffefffeffefffefffeffffeffeffffeffefffefffefffeffffeffefffefffefffggggggggggfffefffffefeffffffffffffhhhe efgffefefffffefffefefffeffffeffefffeffffeffeffffefffefffeffeffffefffefffeffhhgggiigighhgegghfhgehfhhfgghhghhehghg efgeffffeffffefffeffffeffeffefffefffeffeffffeffefffefffefffefffefffeffffeffeffgheggfgehegegeheghfegheheegfggehfhe ffgfeffffeffeffeffffeffefffffeffeffefffffeffeffefffffeffeffeffffeffffeffeffghgfgfegfiefggfegeghgfeghfgeggeghheegf efgfffefefffeffffeffefffefffefffeffffeffffeffeffeffefffffeffefffeffefffefffgeghggggghhgeehhghhhhehghhfghhhhhhhhgg efgfffeeffffffefefffffefefffeffefffefffefffefffefffffefffeffffeefffefffefffgggihghgggfffegegfghegehgefehgghhggehe efgfefffefffefffeffefffefffeffffeffeffffeffeffffeffefffeffffeffefffefffefffggggggggggfffeffffffefefffffffffffhhhe fegfeffefffeffffeffefffefffefffefffeffffeffffefefffefffefffeffffeffeffffeffgghefhiiegeffeggfgegefefefhgggffgfghhh efgfeffffeffeffffeffffefffefefffeffffefefffffeffeffffefeffffeffefffffeffffefffhghfgeffgefgfeehhegeehfegggeghggege eeefeffeffffefffefffeffeffffefffefffefffffeffefeffffefffeffefffefffefffefffffgiieihfeggeegeheegegegfeffggghgfeefe eeefeffeffffeffefffeffffefffeffeffffefffeffefffefffefffefffefffefffefffefffgegffehfgfghffeffeggefegheggfhfgfghegg feeefffefffefffefffefffefffefffefffefffefffefffefffefffefffefffefffefffefffgggggggggggggggggggggggggggggggggggggg efefeffeffffefffefffeffefffefffefffefffefffefffefffeffffeffefffefffefffefffgehhghhiggghhhhhhhhhhhhhhhhhhhhhhhhhhh feefffeefffeffffefffefffffefeffefffefffefffffefeffffeffefffefffeffeffeffeffefeeeefegeefeeeeeeeeegeegeeefeeeggffff efeefffeffeffeffefffefffefffefffefffeffffeffeffffffffffffeffffefefffeffffefhefihehgeefgeehggeefehghfehfehhefhffhf efgffeffeffffeffeffefffeffffeffefffeffffefffeffeffffefffefffffefefffefffffegghihggggfhhgfffhhhheheheehhhhegeghehf efgefffefffefffeffffefefffeefffefffeffeffeffefffffefefefffefffeeffeffefefffeeeeeeeeeeeeeeeeeeeeeeeeeeeeeeeeeeeeee efgfeffeffffefffefffefffefffefffefffefffefffefffeffefffeffffeffefffefffefffggggggggggfffffffffffgfgfggffeffggfggg efgfefffeffffeffeffffeffeffeffffefffefffeffffeffefffefffeffefffefffefffefffgggiiggggggfgggfgfgggefffgfeeefeffggfg ffgfefffffefefffeffefffeffffeffffefeffffefffefffffefefffefffefefefffeffffefefeegfgeeeeehfefeheeegeegeehfgfeeefeee efgffefeffffeffffefffeffefffeffeffffefffeffefffefffefffffeffffeefffefffffefgfiiieeeeeghggghhfghehhghghehgehgghhhe efgffefefffffefffeffeffefffefffffeffffeefffefffeffffefffeffeffffeffefffffefghgghghggggfggfgffgfgefggfggffgfffgfgf efgefffefffeffffeffffefeffffefffeffffefffefffeffffeffeffefffefefefffeffffeffgggfeeeeegfeegeegeeeegeehheeeeheeeffe efgefffeffffefffeffffeffefffefffffefeffeffffefffefffefffeffefffefffefffefffeeeiiehheeeeeeeeeeeeeeeeeeeeeeeeeeeeee efgfffefffefffefffefffefffeeffffeffffeffffefffefeffeffffeffeffffeffefffefffggegfgggggeefefgefehefehhfhhhhhfhhhgfh efgfeffeffffefffefffeffefffefffefffeffffeffefffefffeffffefffeffefffefffefffegggigegeeeeeefegeeeeeeffefefeefffeege fegffeffffeffeffeffefffefffefffefffefffefffefffefffefffffeffeffefffefffefffghihghhihihhgegghghhegfhhggfhhffhhgfhh efgfeffeffffeffeffffeffefffefffefffeffffffeefffefffeffffefffefffeffefffefffgeeiiefieegheegehffhegehfhhehgghgggehe efgfeffeffffffefefffeffeffffffeefffeffffefffeffeffffefffefffeffefffefffefffeeehighhegehfegeheeffefehffhggghfeeeee ffgffeffeffffefeffffeffefffffeffffefefffeffefffeffffeffefffefffffefefffefffegefggefeefgeeefgeheefhgefhegeegeefege efgffeffeffffeffeffefffeffffeffefffeffffefffeffeffffefffefffffefefffefffffegghihggggfhhgfffhhhheheheehhhhegeghehf efgfefffefefffefefffffffefffefffefffefffeffeffffefffeffffefefffefffefffefffgfhihghhgghgfegehffgehehgehfgffgffhehf efgfefffeffffeffeffffefefffefffefffeffffeffffffeffffffeefffeffffeffefffeffffeigfgfeeffhheheheeheeehhehehheghheege efgffeffeffffeffffefffefefffefffffefefffeffeffffeffffeffeffefffefffefffefffgefefheghfghheffghhhefeegfhggfffhhgghh efgfefffeffffeffeffefffefffefffeffffefffffeefffeffffefffefffeffefffeffffefffggihghihghhfehfgffheheghehghhgggggegf efgfefffefffffefefffeffefffeffffeffefffffefefffeeffeffffefffeffefffefffefffghihhfggihhgeeffgffffegegfegeggfffehee efgfeffefffeffffeffeffffeffeffffeffeffeeffffeffeffffefffeffefffeffffefffeffghiggggggfeefeefffgfeeefffffgfffegfffg efgfeffeffffffefefffeffeffffffeefffefffeffffeffeffffffeffeffeffffefeffffffefggfgefhhhegeefgheggegehgggghggggggggh ffgffeffffefffefeffffefefffefffffeffeffffefeffffeffeffffeffefffeffffeffffefgfggggghghfghfegghegegehgggghggggggggh fegffeffffefffefeffffefefffefffffeffeffffeeeffffeffeffffeffefffeffffeffffefgfgggghghfghfeggheggegehgggghggggggggh eegffeffffefffefeffffefeffffefffefffffeffefeffffeffffeffeffefffeffffefffeffgfgggghghfghfeggheggegehgggghggggggggh eegffeffffefffefeffffefeffffefffeffffeffffeffeffeffffeffffefeefeffffefffeffgigggghghfghfeggheggegehgggghggggggggh efgfffeeffffefffefffefffefffeffefffefffeffffffeeffffefffefffefffeffefffeffffggigihgggggeeeehghgeegeehgehffeeghghe eegfffeeffffefffefffefffefffeffefffefffeffffffeefffeffffefffefffeffefffeffffggigihgggggeeeehghgeegeeegeggggeghghe efgfefffffefffefffefeffefffefffefffefffffefefffeffffefffeffefffefffefffefffiiiiiiggihghhggfggfhghgfghfhghghgghghg ffgfeffeffffffeffeffeffefffffefefffeffffefffeffefffeffffefffeffefffefffefffigfihefgghfhggfehgeeefheffffggggghhhhh eegfeffeffffefffeffeffffeffeffffeffefffefffefffeffffeffefffefffeffffeffefffifhiiegihefeeeeefeeeeeegeeeeeeeeeeeeee eegfeffeffffefffeffefffefffefffefffeefffeffefffefffeffffefffeeffeffeffffeffghigfggggggffefegfeffeffgegggggggggggg efgfefffeffffefffeffefffeffefffefffeffffeffefffefffeffffefffeffffefffeffffefegggeffffhggefggegfegegeggeghehehgehh fegfefffefffeffefffeffffeffefffefffeffffeffefffefffeffffefffeffefffefffefffeeghggghggeeeefehfeeeeefeeeeeffefffefe ffgffeffefffffefefffeffeffffefffeffeffffefffeffeffffefffefffeffefffeffffeffhhhfggighihffefggfgfeffgeeffffehgegege feefefffeffffefeffffeffeffffefffefffefffefffeffefffeffffefffeffefffefffefffgffhhgghfeegeeheheegefeggefffgeegfgefg eeffefffefffefffefffeffffeffefffefffeffeffffefffefffefffefffefffefffefffeffghghghgfgfgfgfggfgfggfgfgfggggfggffgfg ffgfefffffffefffefffefffefffefffefffefffefffeffeffffefffefffeffefffeffffeffggfgghfgfgffgffgfgfgegfgfggfggfggggfgg ffgefffffeffeffffeffefffefffefffefffefffefffefffffefefffefffeffeffffefffeffgfgfgfgfgggfggfghfghggfgfggfgfggfggggh fegfeffffeffefffefffeffffeffefffefffefffefffefffefffefffefffeffeffffefffeffgghghhghghgfhgggfggfgfgfgfgfgfggggfgfg ffgfefffefffefffefffeffffefffefffeffefffeffffeffeffffefffeffeffffefffeffffegghghggghgfgfgggggggfgfggfgfgfgfgggggf efefeffefffffefffeffefffefffefffefffefffefffefffefffefffefffeffefffefffefffeeeihehieeffeefegeehfegfhgegffhghgffgf feefefffefffefffeffefffefffefffeffffefffffeffefefffeffffefffeffffefefffeffffefegfhhfeefeefefeefeefffeeeeeeefgfege efgfefffeffffeffefffeffeffffeffefffeffffeffefffefffefffeffffeffefffefffefffihghgehfhegffeeegffeeggfeeggfghfeehhhf efgfefffefffefffefffeffefffefffefffefffefffefffeffffffefefffffeffeffeffffefghhgigefhhfhgefehhffehfhgehehhfheghehe ffgffeffefffefffeffefffefffefffeffffefffeffefffffefefffeffffeffffeffefffeffiegifgeggfeefegfgefffegegfegegghggfegg efgfefffefffffeffefffeffeffffefffeffefffffeffefeffffefffeffffefffeffefffeffgehhgghgfgggfehehhhfehghffhfffgheffggh ffgfeffeffffeffffeffeffeffffefffeffeffffffeefffefffefffefffefffefffefffefffeegfhgggggffffffffffffffffffffffffffff eegefffeffffeffefffefffefffefffefffeffffeffefffefffefffeffffeffeffffeffefffgfgihehigeeeeeeeeeeeeeeeeeeeeegeeegeee efgffeffefffeffffefffeffeffffeffeffeffffefffefffeffffeffefffeffffefefffffefgghhhgghfgggfegfgfffegefgegffffgghgfgf efgfefffefffffeefffefffefffefffefffeffffeffefffefffeffffefffeffeffffeffeffffggffgighgfgeeffgefhffffffffffffffffff efffefffffefeffffeffeffefffefffeffffefffeffefffeffffefffeffefffefffefffefffhggghgihgffghegehggggghfghfggfgggfgfgg efgfefffefffffefefffefffefffeffffefffeffefffeffffefeffffffeeffffefffffefefffegigfgegfefeeeeeffgeffffeggefgggeeeef eegfefffefffefffefffefffefffefffefffeffffeffefffefffeffffefeffffefffefffeffeeeeeefegeeefefgeeefefeeeeffeeefffffff ffgfeffeffffefffeffefffefffefffefffeffffefffeffefffeffffeffefffefffefffefffgegigeiigeehgegehegffegehgegehghghgehg efgfefffeffffefffefffefeffffeffffeffefffeffffeffefffeffefffefffeffffefffeffhegfggfgggeheehehgghfhffgfhfehfhhggfhe efgfffefffefffeffefefffefffefffefffeffffffeefffefffefffffefeffffeffefffefffgfgfffefegffeeeeffgfeeegfegeeefefefege efgfeffffeffeffffeffeffeffffffeeffffeffeffffffeeffffeffefffffeffffeeffffeffifigegfifhheeeehegggfgggfeffgfeegggeff ffgfeffeffffefffefffeffefffefffefffefffefffefffefffeffffeffefffeffffeffefffehgfgeffehhheefehhgefeegeegefgeheegefe efgeffffefffeffefffefffefffefffefffefffefffefffefffefffeffffeffefffefffefffeeeeeeeeeeefeeeeeeeeeeeeeeeeeeeeeeeehe efgfefffffeffefeeffefffefffeffeeefffefffefeefffefffeffffeefefffefffefffefffgfgfhggheefgeehehfggeeeggeheffgghghege efgefffefffefffefffefffefffefffefffefffef fefffefffefffefffefffefffefffefffggggggggggffggffggfggfgggffffgggfggfff ffffeffefffffefefffffefffeffeffeffffefffefffefffefffefffefffeffefffeffffeffihggggghhgeffeefeeefehfheefffgfgehhfhf feeefffeffffeffefffefffefffeffffefffefffeffefffeffffeffefffefffefffefffefffgegiifeifefgeegfheegegegfegffgfefggefe ffgfefffefffeffeffffeffffefefffefffefffefffefffefffefffeffffefffffffeffefffgghigghghhffgeeefegfefeegffeeehheegehe efgfeffeffffefffefffeffefffefffeffffeffffefffefefffeffffefffefffffeffefgghfgghfgfggfefgeegegeegeheggegggegffgeegf efgfeffeffffffeefffefffefffefffefffeffffffeefffefffeffffefffeffefffeffffeffgegigegihehheegehgegeeeghehhhehheheehg efgfefffffeffeffeffffefffeffeffeffffeffffeffeffffefffeffeffeffffeffefffefffghffggfhefgeefeegfehgehfehgefgeghffeee efgfefffefffeffeffffefffefffeffffeffefffeffffeffefffeffeffffeffefffeffffeffgefhifegefhgeeeefeggeghgfegfehegfgefgf efgfeffeffffffeefffefffefffeffffffefeffefffeffffeffefffefffefffefffefffefffggfihehhgeeeeeeeeeeeeeeeeeeeeeeeeeeeee ffgffefffeffffefffefeffeffffefffeffffefffefffeffeffffefffeffeffffefefffffefgggheiiiiihghegfhghheeeefehghhhhehgeff efgfffefefffffeeffffffefefffefffefffefffefffefffefffffeffeffeffeffeffefffefgggggfefegefffeeeffefffffeeeeeehfffgfg efgfeffeffffefffeffefffefffefffefffefffefffefffefffeffffeffefffefffefffefffeeghgfffeeeeeeeefeeeeeeffegeeeeeeeeege efgfefffefffeffffeffefffefffeffeffffefffefffefffefffefffefffefffefffefffeffghghghghgggfgghggggghgggggfgggfggggfgg ffgfeffffeffeffffeffeffffeffefffeffffefffefffeffefffefffefffeffeffffefffeffghghghgfgggfgfgggfgfggggggfggfgfggggfg efgfefffefffeffffefffefffeffefffeffffeffeffffeffefffefffefffefffefffefffeffgfgfgggfgfffggfgfgfggfgfgffgffgfggghgh ffgfefffefffefffeffffeffefffefffefffefffefffefffefffefffefffefffefffefffeffgfgfghgfgggfgggfgfgfgfgfgfgfggfgfgfggg ffgfeffffefffeffffeffeffefffefffefffefffefffefffefffefeffefefffefffeffffeffgfgfgggghggfgfgfgggggfgfgfgfgffggggfgf ffgfefffefffefffefffefffefffefffefffefffefffefffeffeffffefffeffeffffefffeffgfgfggggfgfgfgfgfggggfgfgggfggfgfgggff eegfefffefffeffffeffefffefffefffefffefffefffefffefffefffefffefffeffeffffeffgfgggggfgggfggfgfgfghfggfggfggfgggggfg ffgeffffefffefffefffeffffeffeffefffeffffefffefffefffefffefffefffefffefffeffgfgfgggfhggfgfgfgggggfgggghfgfgfggfgfg ffgeffffefffefffeffffeffefffefffefffefffeffffeffeffefffefffeffffefffefffefffgfgfggfgggegfgfgfggggghfgfgfggfggggfg eegeffffeffffefffeffefffefffefffefffefffefffefffefffeffefffefffeffffeffffefgggfgggfgfgffgfgfgfggfgfgfggggfggggggf ffgeffffeffffeffefffeffffefffefffeffefffefffefffefffefffeffeffffefffefffefffgfgfggfgggfgfgfgfggegfgfeggfgfgfgfgfg efgfefffeffffeffefffeffffeffefffeffffefefffefffeffffefffeffefffeffffffefeffggggggggggffffffffffffffffffffffffffff efgefffeffffefffefffeffeffffffefeffefffefffefffefffffeffeffffefffeffeffffefgggggfgggggggggggggggggggggggggggggggg effffefefffffefeffffefffeffffefffeffefffefffeffefeffefffeffefefefeffefffeeggggggggggefghefghhhefehhhheehehgggggg fegffefeffffefffefffefffefffeffefffefffffefefffeffffeffffeffefffeffefffefffgghfggfheeggeefegeefegfgffgefgegfegffe eegfeffefffffeffeffefffeffeefffefffeffffffeefffefffeffffefffeffefffefffefffeehhgeehfgefeeeegeeeefefeefeeeeeeeeeee eegffefefffffeffeffefefeffeefefefffefffeeffeeffeeffeeeeeeeefffeffefeffeeeffgfeiigihhghggeheefhheegggggfgfghhhhhhe efgffeffffefffefeffefffeffffffeefffeffffffeefffeffffefffeffefffefffefffefffgehhggghgghhgeeegggfefegefffeffggfgegf ffgfeffffeffefffffeffeffeffefffefffffefffeffefffeffffeffffefefffeffffeffeffggghhgefgghhgggghghhhghgeeefghghhegghh efgfefffeffeffffeffeffffeffefffefffefffefffefffeffffefffefffeeffeffefffefffghgfgeeggeefehhhhggeeeeeheghhegheggghh ffgfefffefffffefefffefffeffeffffefffefffffeffeffefffeffffeffeffeffffeffffefghgfghhgfgggfehegeghegggheghhghhhhggge efgfefffefffeffffeffeffffeffeffffeffeffffeffffefefffefffeffffeffefffeffffefggfghgfgeefgfegfhffggegfhgffefgghfgghe ffgfeffeffffeffefffefffefffefffffefefffeffffeffefffefffefffefffefffeffffeffggihggeeeehfeefehfhfefehhehhheehfhhehf efgffefffefeffffeffffeffefffffeffeffeffeffffffefefffefffefffeffeffffefffeffeghfigfgfhefgfeghfgfefghfefegfhgfhfgfh efgfeffefffffeffeffefffefffeeffefffeffffeffefffefffeffffeffefffeffffefffefffeghheihfhfgfefegffeegehfegeggefgggehe efgffeffffefffefeffeffffeffffeffeffeffffffeefffefffffeffeffffefefffefffffefghgfghgfghffggefgeeehhghfgfgffgfhheehe efgeffffeffffefeffffeffefffefffeffffefffefffeffeffffefffffeefffefffefffefffgegfgeghffhhgeheegfhfgfggfgghgfhgggfhf efgfefffefffefffefffefffefffefffefffeffefffeffffefffeffefffffefefffeffffeffggggggggggfgfffgfgfgffgfffggfggffgffgg ffgefffefffeffffeffeffffefffeffefffeffffeffefffeffffeffeffffefffeffefffefffghgggghgggffgffgggffffggfffgffgfgffggf efgfeffefffffeffeffeffffefffefffefffeffffeffefffefffefffeffefffefffffefefffgfgfhffffghgfeeefeeeefefeefghghgggfghg efgfffefffefffefeffefffeffffffeefffeffffffeefffefffffefffeffffeefffffefffefiggiieiigghheegfhghhehfhhehhhhhhhhhhhg eegfefffefffffefefffeffeffffeffefffeffffeffeffffefffefffeffffefefffffeffeffgegifgfighfhgeffhfefefehfefefggggfgfhe efgeffffefffffefefffefffeffffeffefffeffffefffeffefffeffffeffefffeffefffeffffhfffhhgghhhgehhhehheeefheheheheeehhhe ffgfeffffeffffeeffffefffeffefffefffefffefffefffefffeffffeffeffffeffeffffefffgeiggegigfhhefggfffefeheefgffhhffhehf efgfeffffefeffeffefffeffefffefffeffffefeffeffefffeffefffeffeffffefffeffffefggggggggggffgggfggfgeffffffefgfefeffff ffgfefffefffeffffefffefffeffefffeffffeffefffefffefffefffefffefffefffefffeffgfgfgggfgfgfgfgfggfggfgfgfgfgfgfgggfgf ffgfffefefffffeeffffeffefffefffeffffffeefffefffeffffefffeffefffefffefffefffggghhehggggheefefgffegefffgfghhhfgfegf efgfefffffeffeffefffeffefffefffeffffffeffeffefffeffffefeffffeffeffffefffeffgggggggggefheefeggfgegeffgfffgfggfggfh ffgfeffeffffeffeffffeffefffefffefffeffffeffefffefffefffefffefffffefefffefffgegiieiieehhgehehehhehehhehhhhhhhhhfhh eegfeffefffffeffefffeffefffefffeffffffefeffeffffefffefffefffeffefffffeffeffghgffegfgefgggfggfffgfffgfgggggfgggggg fegfeffffeffeffffefffefffeffefffffefefffffefefffffefeffffeffffeffeffeffefffgfggehgfggghfegegegfgffffgehgggggfgggh eeffefffefffeffffefffeffffeffeffffeffefffeffefffefffefffeffeffffefffeffffefegffggffgeffgggffgfeeeeggehhggfggfggge eegfffefefffefffffeffeffefffefffefffefffeffffeffffefffeffeffefefffefffefffegffeeeffhhffgfeffgfeefggfggggfgffefgfe eegfefffefffefffefffffefefffefffffefffeffeffefffefffefffefffeffffeffefffefffgfgggfffeffffeeehhhgfeefggfeegghhhgge eegffeffeffffefffeffffefffefffefffeffeffefffffefefffeffffefeffffefffefffeffeffggggghihhhefffhhhggheeeeghghghhgghh efgfefffefffffeffeffeffefffffeffffeffeffffeeffffefffffeffefefffefffefffefffeegiieihhehgfeehhfefehehfehfhhfhfhhfhh efgfeffffeffeffefffefffefffefffefffeffffffeefffefffffeffffeffeffefffffeffefhfihfiffhfgheeffgfheehghefhfhhfgehhgge eegfeffefffffeffeffffeffeffefffffeffeffffeffffefefffeffffefeffffeffefffefffgghfhgggghfghfegffgeefghgfeggfgegfgfge ffgfffeffeffffeffeffeffeffffeffefffefffffefffeffefffeffeffffeffefffefffefffiggiiggfhffgefghfggehgehefehfefhhfeeeh efgfefffeffffeffefffefffefffeffefffefffffeffefffefffefffeffffeffeffffefffefggghhggghgefheeffefhefehhefhfhhhhhhehh efgffefeffffefffefffeffeffffeffefffeffffeffefffeffffefffeffffeffeffefffefffgegheeeggefggfggggggfgggggfggggggggggg eegfeffeffffefffeffeffffeffefffefffefffeffffeffefffeffffefffeffefffefffeffffegihghiggefgeghgfffeeeghegfffehffgehf efgfefffeffffeffefffeffefffefffffefeffffefffffeefffeffffeffefffeffffeffefffgfgfgffgfeefgeeefffefeegfeffeffefefffe fegfefffffeffeffeffefffefffeffffeffefffffefffefeffffffeeffffefffefffefffeffgggggggfhgggfefeegegehfggegggggggghfhf eegffefffeffffefffefffeffeffffefffeffefffeffffeeffffefffefffefffeffffeffeffghfghifgeihhggghhhhheghghghggeghhhgfge efgfeffeffffefffeffefffefffefffefffeffffffeeffffefffefffeffeffffeffeffffffegggghehheehhhegfhfhgegegfefefgggfegfge eegfefffeffffefefffefffefffefffefffefffefffefffefffeffffeffeffffeffefffefffhffihehhfeefeeeegeeeeeehfeeeeeeeeeeeee ffgfeffeffffefffefffeffefffefffefffefffefffefffeffffefffeffefffefffefffefffgggggfggfgeffefffhffeffhffhfhffhffhfhf ffgffeffffeffeffefffeffeffffeffffeffefffeffffeffeffffeffefffefffefffeffffefiiigggggggfggfggggggeffeggggffgffggggf efgffeffefffefffefffeffefffefffefffeffffeffefffeffffefffefffffeeffffeffffeffggiihgihghhgefhhghhegehhehhhhehhhhehg ffgfefffeffffeffefffeffefffeffffefffeffefffefffefffeffffefffefffeffffeffeffggfgfggffggfghhhghghghghgfhghghghghgeg efgffeffffefffefffeefffefffeffffeffefffefffefffeffffeffeffffefffeffffeffffegeegeegegehfgefheegfgeefgehfhegfgeffhe fegfefffffeffeffeffefffeffffeffefffefffeffffeffefffffeffeffeffffeffffefffefghgefgegffefegefffeggegffhegfefegeffeg ffgfeffeffffefffeffefffefffefffffeffefffefffeffeffffefffeffffeffefffefffeffgggffgffggfgffffffgfefeefeeffeefefefef ffgffefeffffffefefffeffefffefffeffffffeefffeffffefffeffefffefffefffefffefffhehiiehiheefeeeeeeegefeegegeefeegeeehe efgfffefeffffeffefffefffeffffefffefffeffeffffeffefffefffeffefffeffffefffeffggegegggegegeeggfgfggfgefgfggffggfgfgf efgffeffeffffeffefffeffefffefffeffefffefffefffefffefffffefffeffefffefffefffgghgghgfghfgegefgefgfgggfgfgfegefgfgfg efgffefeffffffefeffefffefffffefefffeffffffeefffefffffeffffeffefefffffefffefhhhffhffhffefeeffefeegggefgefggfeffgfe**
