# Supplementary material for: Coping, health anxiety, and stress among health professionals during Covid-19, Cape Coast, Ghana
Source: PLoS One. 2024 Jan 29;19(1):e0296720. doi: 10.1371/journal.pone.0296720 (PMC10824413; doi:10.1371/journal.pone.0296720)
Supplement: Attachment — Submitted filename: Response to reviewers .docx [file pone.0296720.s002.docx]

Dear Pierfelice Cutrufelli

Reviewer #1:

1. Ethical Considerations: Your manuscript currently lacks sufficient detail in relation to how participant confidentiality was assured.

Response: Participant confidentiality assured revised.

2. Data Accessibility: More precise information about how and where the data can be accessed would greatly enhance the transparency of your study.

Response: Data accessibility included.

3. Recruitment: The clarity of your procedure is somewhat lacking. While it appears that you have employed the optimal Multistage Sampling technique, the recruitment procedures outlined are presented in a vague and confusing manner. Furthermore, the absence of a comprehensive demographic report hinders our ability to determine the congruence of the sampling approach.

Response: Demographic report included. Table 1

4. Scales used: The utilization of two stress scales warrants proper justification to enhance clarity and academic rigor. It is imperative to provide a more thorough explanation regarding the rationale behind the chosen approach. It is important to bear in mind that the subsequent discussion should extensively analyze and highlight the disparities stemming from the utilization of these two stress scales.

Response: Justification made and discussion made

5. Results: There seems to be an omission of results concerning gender differences analyses, despite it being one of your main hypotheses. Furthermore, the data presented in Table 3 raises concerns. Despite text suggesting that positive reframing (β = .143, p = .051) significantly contributes to health anxiety, the p-value reported exceeds the conventionally accepted threshold of significance (typically set at .05 or lower). In addition, it is worth noting that data interpretation occasionally appeared to be interpolated in the presented findings, as it should ideally be situated within the discussion section.

Response: Omission included and data in Table 3 Corrected. Positive reframing and other errors corrected.

6. Comprehensive Reporting: For the benefit of a thorough interpretation of your study, ensure all data analyses and relevant demographic information are included in your report in a detailed manner. For instance, demographic data differentiating nurses from other health professionals in each facility, along with details on work experience / age for these groups, should be included to offer a more complete understanding of your findings. By presenting all demographics and relevant analyses, you can avoid selective reporting, thereby enhancing the validity and robustness of your results.

Response: relevant demographic information included

7. Discussion: The clarity and coherence of your discussion section could be improved. The implications and significance of your hypothesis need to be more explicitly addressed, as the current explanation of the regression data is unclear. In addition, reference to prior studies that provide a broader context to your findings could be beneficial. It is evident that some of the referenced citations do not adequately support your hypothesis or clarify the points you are making. You should also explicitly state the correlational nature of the study design and the limitations inherent in establishing causality. For example, considering potential bidirectional relationships and unaccounted variables that may influence the observed associations, such as the potential confounding factor of the female gender and nursing profession, could enhance your discussion. You have sometimes made unsupported claims based on your data in the discussion, and that should be revised.

Response: corrections effected and other errors deleted .

8. Study Limitations: An explicit discussion addressing the limitations of your study, such as potential recruitment biases or issues of generalizability, is recommended. Incorporating this information will contribute to strengthening your paper's reliability.

Reponses: Limitations of the study addressed.

9. Recommendations: Strengthen your recommendations section by suggesting more specific strategies for implementing your findings in clinical practice. This will make your recommendations more useful and applicable.

Response: Recommendations strengthened.

10. Conclusion: Your conclusion would benefit from more explicit suggestions for future research, derived from your own findings. This will provide clearer guidance for future investigations in this field. Avoid also here unsubstantiated claims (see point 7).
E.g. The interpretation of the data you proposed is incongruent with the obtained results: the absence of correlation does not necessarily indicate a positive impact.

Response: Corrections effected.

11. Readability and Language Quality: I recommend a comprehensive proofreading to enhance readability and rectify any English language errors. The current state of the text exhibits a certain degree of compromised fluency, thereby necessitating and affording an opportunity for improvement.

Response: Comprehensive proofreading made

Dear Antonio Di Francesco

Reviewer #2: 

 COPING STRATEGIES AS PREDICTORS OF HEALTH ANXIETY AND STRESS AMONG HEALTH PROFESSIONALS DURING COVID-19 PANDEMIC IN CAPE COAST METROPOLIS, GHANA


Firstly, while the topic of your study is of interest, the manuscript does not seem to add significant new information to the existing literature. The positive association between the implementation of coping strategies and levels of stress and hypochondria is already well-documented.

Response: Significant information added to the literature. Topic of the paper amended due to some errors made in the analysis.

Secondly, the manuscript contains numerous formatting errors that make it difficult to follow. The language used needs to be improved to meet the standard expected for a scientific paper. There are instances of unclear or awkward phrasing that detract from the overall readability of the text. I would strongly recommend a thorough proofreading and editing of the manuscript to improve its clarity and readability.

response: comprehensive review and proofreading made

Furthermore, there are missing data points in your manuscript, which further hinder the comprehension of the study's findings. Please ensure that all necessary data are included and clearly presented.

Response: Addressed

In light of these issues, I recommend a thorough revision of the manuscript. Addressing these concerns could significantly improve the quality of your work and its suitability for publication.

Response: Concerns Addressed
