# Supplementary material for: Coping, health anxiety, and stress among health professionals during Covid-19, Cape Coast, Ghana
Source: PLoS One. 2024 Jan 29;19(1):e0296720. doi: 10.1371/journal.pone.0296720 (PMC10824413; doi:10.1371/journal.pone.0296720)
Supplement: Attachment — Submitted filename: Response to the academic editor.docx [file pone.0296720.s003.docx]

Response to the academic editor,

Thank you for your efforts to improve my manuscript to meet PLOS ONE standards. Being guided by the comments made by the academic editor, I have improved the data implication and its relevance to the field. I have also engaged additional language editing services to help me improve on the manuscript. I will be grateful if my manuscript fits the standard for approval and publication.
